# Psychological disorders and suicide attempts in youths during the pre-COVID and post-COVID era in a Taiwan pediatric emergency department

**DOI:** 10.3389/fpsyg.2023.1281806

**Published:** 2023-10-16

**Authors:** Bei-Cyuan Guo, Ying-Ju Chen, Wun-Yan Huang, Mao-Jen Lin, Han-Ping Wu

**Affiliations:** ^1^Department of Pediatrics, National Cheng Kung University Hospital, College of Medicine, National Cheng Kung University, Tainan, Taiwan; ^2^Department of Rehabilitation, New Tai-Ping Cheng Ching Hospital, Taichung, Taiwan; ^3^Department of Pediatric Emergency Medicine, China Medical University Children’s Hospital, China Medical University, Taichung, Taiwan; ^4^Department of Medicine, Taichung Tzu Chi Hospital, The Buddhist Tzu Chi Medical Foundation, Taichung, Taiwan; ^5^Department of Medicine, College of Medicine, Tzu Chi University, Hualien, Taiwan; ^6^Department of Pediatrics, Chiayi Chang Gung Memorial Hospital, Chiayi, Taiwan; ^7^College of Medicine, Chang Gung University, Taoyuan, Taiwan

**Keywords:** COVID-19, psychological disorders, psychological fragility, suicidal attempt, overdose, emergency department, pediatric patients

## Abstract

**Background:**

The COVID-19 pandemic has had a significant impact on pediatric patients, increasing their vulnerability to psychological fragility. The aim of this study was to investigate the epidemiology and clinical spectrum of pediatric psychological fragility and suicide attempts in the emergency department (ED) before and after the onset of the COVID-19 outbreak.

**Methods:**

A total of 340 pediatric patients admitted to the ED for psychological fragility between 2019 and 2022 were retrospectively collated and categorized according to three periods: pre pandemic, pandemic, and post pandemic. Epidemiological and clinical information were analyzed and compared among the three groups. Moreover, patients with suicidal ideation or suicidal attempts and types of substance use disorders in children with suicidal attempts sent to the ED were analyzed.

**Results:**

The proportion of psychological fragility increased during the pandemic period (0.4%) and the post-pandemic period (0.8%) compared to that in the pre-pandemic period (0.28%). Suicide ideation was the highest before the pandemic period (0.04%), while suicidal attempts were the highest in the post pandemic period (0.42%). Significantly elevated trends in suicide attempts involving overdose and injury were observed among the three groups (*p* < 0.05). Intensive care unit (ICU) admission rates increased significantly after the COVID-19 outbreak (*p* < 0.05), and major depressive disorder was the most common psychological fragility in the ED in all three groups.

**Conclusion:**

An increase in the proportion of pediatric psychological fragility in the ED was noted in the post pandemic period than before or during the pandemic. With higher rates of ICU admissions and an increase in suicide attempts among children and adolescents during the pandemic compared to before or after the pandemic, it is of utmost importance to provide mental health support to this vulnerable population in order to prevent suicide attempts in the event of a new global outbreak of infectious diseases.

## Introduction

The Coronavirus disease 2019 (COVID-19) outbreak was declared a pandemic by the World Health Organization in March 2020 (Machhi et al., 2020; World Health Organization, 2020; Zhou et al., 2020), while the Taiwan Center for Disease Control classified it as a Category 5 communicable disease in January 2020. The COVID-19 pandemic has wreaked havoc worldwide, killing millions of people with approximately 18% of children under the age of 18 years comprising the American Academy of Pediatrics (2023). The government’s policy forced billions of people into quarantine to prevent the spread of COVID-19. Owing to social isolation and concerns about contagion, the mental health consequences associated with the COVID-19 crisis are worsening. Globally, 10–20% of teenagers suffer from mental health problems (Binagwaho and Senga, 2021), and the statistics may be influenced by their vulnerability during the pandemic. Behavioral changes and early psychological distress associated with the COVID-19 pandemic have been observed in both children and adolescents (Uccella et al., 2021; Profio et al., 2022). Disruption of social life and important activities caused stress among children and adolescents during the COVID-19 lockdown (Cianfarani and Pampanini, 2021), and they may experience mood or anxiety symptoms due to these quarantine measures (Segre et al., 2021). Teenagers may be lacking the willingness and knowledge to seek help for this new and big challenge (Hellström and Beckman, 2021). Poor mental health among adolescents is associated with negative outcomes, such as suicide, behavioral problems, and emotional distress. Globally, China in Asia and Italy in Europe were among the first countries to be significantly affected by the virus. During the COVID-19 pandemic in China, children of all age groups exhibited severe psychological conditions, such as clinging, inattention, and irritability. Interestingly, differences in psychological conditions were observed between the younger age group (3–6 years), which showed symptoms of clinginess and fear, and the older age group (6–18 years), which displayed symptoms of inattention and persistent inquiry (Jiao et al., 2020). Another study conducted in China found higher levels of depressive symptoms in regions affected by the COVID-19 outbreak compared to areas that were not affected (Xie et al., 2020). During the COVID-19 pandemic in Italy, there was a significant increase in patient admissions for neuropsychological disorders (NPD) during the COVID-19 pandemic. The two most frequently observed NPD conditions that increased were suicidal ideation and depression. Additionally, there was an observed increase in hospitalizations related to neuropsychological disorders (Bozzola et al., 2022). In Taiwan, government isolation and limiting policies were implemented starting from late January 2020, shortly after the first COVID-19 patient was identified in Taiwan. These measures may have caused psychological fragility in both children and adolescents; however, these is limited research on this topic in Taiwan. Children and adolescents experiencing psychological fragility can seek assistance at the pediatric emergency department (ED). However, the clinical presentation and epidemiology of these disorders may have changed during the pandemic. Therefore, this study aimed to compare the epidemiology and clinical characteristics of psychological fragility among children and adolescents admitted to the ED before, during, and after the COVID-19 outbreak in pediatric emergency in Taiwan.

## Materials and methods

### Patient population

This retrospective chart review was conducted in the pediatric ED of a medical center in Central Taiwan between February 2019 and January 2022. Patients were included in the study if they presented primarily for a behavioral health concern. Additionally, patients were also included if there was any mention of a psychiatric problem in their chief complaint or ICD-10 diagnosis. The diagnosis of psychological fragility was typically confirmed through the original ICD-10 diagnosis or consultation with a psychiatrist. All included pediatric patients were divided into three groups based on the period of visit: (1) Group 1 (pre-pandemic period, February 2019–January 2020), Group 2 (pandemic period, February 2020–January 2021), and (3) Group 3 (post-pandemic period, February 2021–January 2022) Subsequently, the data of patients who visited the ED were collected for further analysis. This study was approved by our Institutional Review Board of China Medical University Hospital (CMUH110-REC3-083). All methods were performed in accordance with the relevant guidelines and regulations. Data were collected, reviewed, de-identified, and anonymized before analysis. The ethics committee waived the requirement for informed consent because of the anonymized nature of the data and the scientific purpose of the study.

### Study design

Clinical data including age, sex, definite diagnoses, categories of major psychiatric diagnosis, disposition, initial level of triage, reasons for psychiatric consultation, number of patients with suicidal ideation, reasons for suicide attempt, type of drugs, methods of suicide, trend of patients (discharge, admission, patient expired, or discharge against medical advice), duration of ED stay, admission rate, length of hospital stay, intensive care unit (ICU) admission rate, length of ICU stay, mortality rate, emergency surgery, and PED revisits within 72 h were gathered from the medical records. Primary discharge diagnosis codes based on the International Classification of Diseases, tenth revision (ICD-10) were used for categorization and were defined by psychiatrists. All clinical factors were analyzed and compared among pediatric behavioral health patients in the three groups. Moreover, we analyzed patients with suicidal ideation or suicidal attempt sent to the ED among the three groups, and analyzed in detail the types of substance use disorders in children with suicidal attempt.

### Statistical analysis

Categorical variables were analyzed using the chi-squared test or Fisher’s exact test, as appropriate. Continuous variables were analyzed using the t test or Mann–Whitney test. In the descriptive analysis, values were presented as numbers, percentages, median (IQR), and mean ± standard deviation (SD). value of ps <0.05 were considered statistically significant. All statistical analyses were conducted using IBM SPSS Statistics software (version 22.0; SPSS Inc., Chicago, IL, United States).

## Results

### Demographics

**Figure 1:**
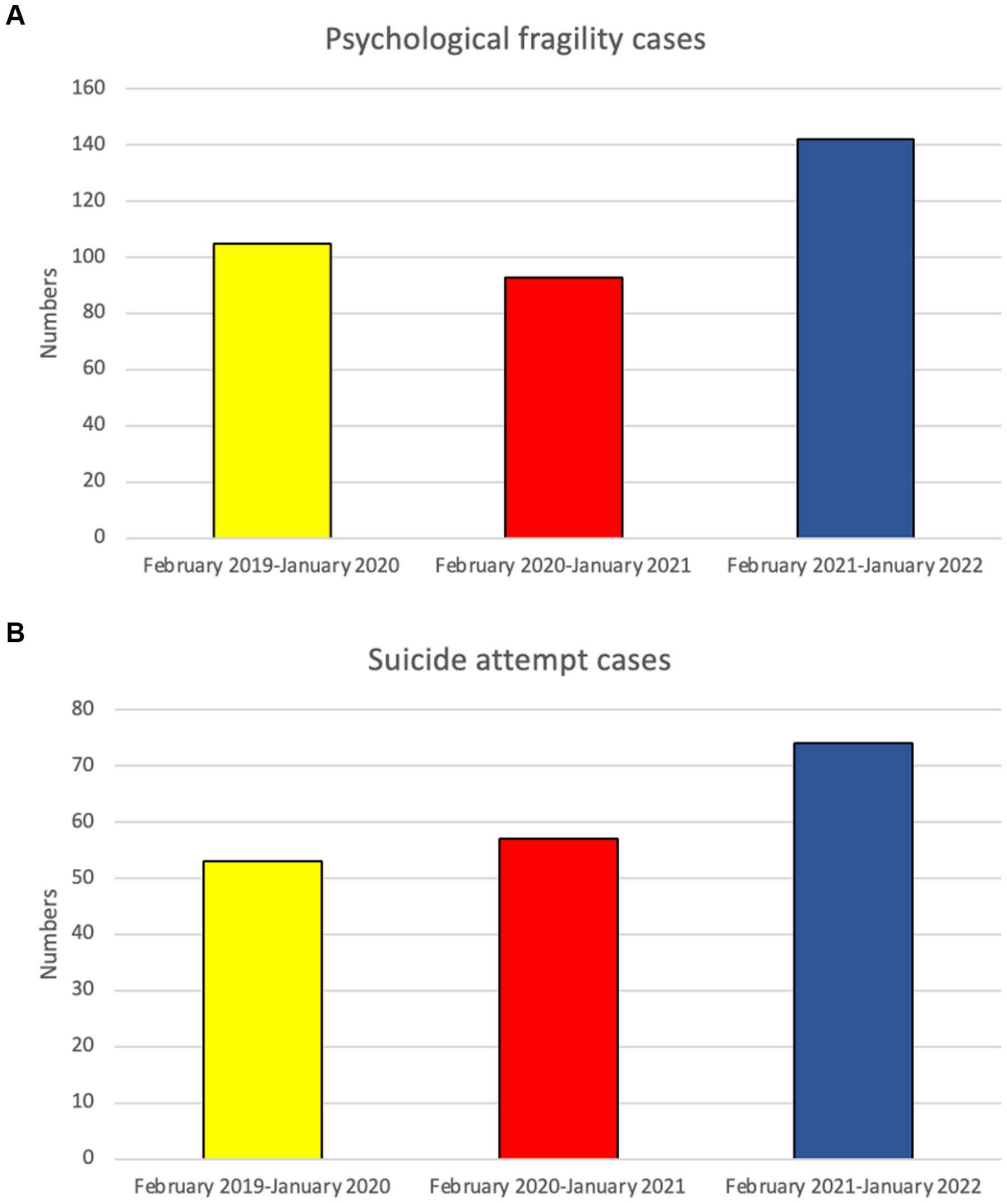
The numbers of psychosomatic pediatric patients **(A)**; the numbers of children with Suicidal attempt **(B)** admitted to the ED in the 3 groups.

During the 3-year study period, 77,955 pediatric patients who presented at the pediatric ED were enrolled in this study. Of all the patients, 37,097 were categorized into Group 1; 23,028 into Group 2; and 17,830 into Group 3. However, these numbers have decreased annually. During the pandemic period, there was a notable trend of shorter length of stay in the pediatric emergency room for observation across all age groups, with a significant change in the infant and school-age groups [both *p* < 0.001 (Table 1)]. In total, 340 pediatric patients with psychological fragility visited the ED, including 105 patients in Group 1, 93 in Group 2, and 142 in Group 3 (Figure 1A). During the pandemic and post-pandemic periods, pediatric psychological fragility accounted for 0.40 and 0.80%, respectively, in all the ED visits, which was more than pre-pandemic period (0.28%). In addition, 15 pediatric patients sent to the ED had suicidal ideation in Group 1 (0.040%), 8 in group 2 (0.035%), and 3 in Group 3 (0.050%) while 53 patients were cases of attempted suicide in Group 1 (0.14%), 57 in Group 2 (0.25%), and 74 in Group 3 (0.42%).

**Table 1:** Comparison of clinical characteristics of patients with psychologic diseases in the pediatric ED among the 3 groups.

Variables	Group 1	Group 2	Group 3	*p* for trend
	(*n* = 105)	(*n* = 93)	(*n* = 142)	
Sex male	23 (21.9)	32 (34.4)	49 (34.5)	<0.001***
Female	82 (78.1)	61 (65.6)	93 (65.5)	<0.001***
Triage level				
1	1 (1.0)	4 (4.3)	4 (2.8)	0.0299*
2	38 (36.2)	44 (47.3)	59 (41.5)	<0.001***
3	57 (54.3)	38 (40.9)	72 (50.7)	<0.001***
4	9 (8.6)	5 (5.4)	6 (4.2)	0.588
5	0 (0)	2 (2.2)	1 (0.7)	0.2101
Age				
9–12 y	8 (7.6)	13 (14.0)	9 (6.3)	0.0104*
13–15 y	43 (41.0)	35 (37.6)	66 (46.5)	<0.001***
16–18 y	54 (51.4)	45 (48.4)	67 (47.2)	<0.001***
Consult psychiatry				
Psychological fragility	69 (65.7)	65 (69.9)	85 (59.9)	<0.001***
Male	9 (8.6)	17 (18.3)	18 (12.7)	
Female	60 (57.1)	48 (51.6)	67 (47.2)	
Emotion problem	36 (34.3)	28 (30.1)	57 (40.1)	<0.001***
Male	14 (13.3)	15 (16.1)	31 (21.8)	
Female	22 (21.0)	13 (14.0)	26 (18.3)	
Suicidal ideation	15 (14.3)	8 (8.6)	9 (6.3)	0.6739
Suicidal attempt	53 (50.4)	57 (61.2)	74 (52.1)	<0.001***
Drug overdose	40 (38.1)	51 (54.8)	44 (31.0)	<0.001***
Injury	40 (38.1)	35 (37.6)	59 (41.5)	<0.001***
Suicide injury				
Cutting	22 (21.0)	21 (22.6)	26 (18.3)	0.0016**
Hang up	0 (0)	1 (1.1)	0 (0)	0.758
Jump down building	3 (2.9)	1 (1.1)	5 (3.5)	0.0792
Burning charcoal	0 (0)	0 (0)	1 (0.7)	0.12
Drink detergent	1 (1.0)	0 (0)	1 (0.7)	0.663
Mortality rate				
Sustained OHCA at PED	1 (1.0)	0 (0)	1 (0.7)	0.663
Overall mortality rate in ICUs	0 (0)	0 (0)	1 (0.7)	0.12
Location				
MBD	57 (54.3)	54 (58.1)	87 (61.3)	<0.001***
Expired	1 (1.0)	0 (0)	1 (0.7)	0.663
AAD	21 (20.0)	8 (8.6)	24 (16.9)	0.0058**
Admission	26 (24.8)	31 (33.3)	30 (21.1)	<0.001***
Admission				
ward	26 (24.8)	25 (26.9)	26 (18.3)	0.0068**
ICU	0 (0)	6 (6.5)	4 (2.8)	0.0107*
Length of hospital stay				
In ward, median (IQR), days	8.5 (8.1)	2 (2.2)	6 (4.2)	0.001***
In ICU, median (IQR), days	0 (0)	2.5 (2.7)	5 (3.5)	0.388
PED observation time, median (IQR), *h*	5.2 (5.0)	3.9 (4.2)	5.15 (3.6)	0.3373

The value of *p* is calculated by comparing the number of patients with psychological diseases to the total number of visits to the pediatric emergency department.

#Cochran-Armitage test; value of ps, *p < 0.05; **p < 0.01; ***p < 0.001.

OHCA, out-of-hospital cardiac arrest; PED, pediatric emergency department; ICU, intensive care unit; ROSC, return of spontaneous circulation; IQR, interquartile range; MBD, may be discharge; AAD, against advice discharge.

### Comparison between behavioral health pediatric patients in the three groups

**Figure 2:**
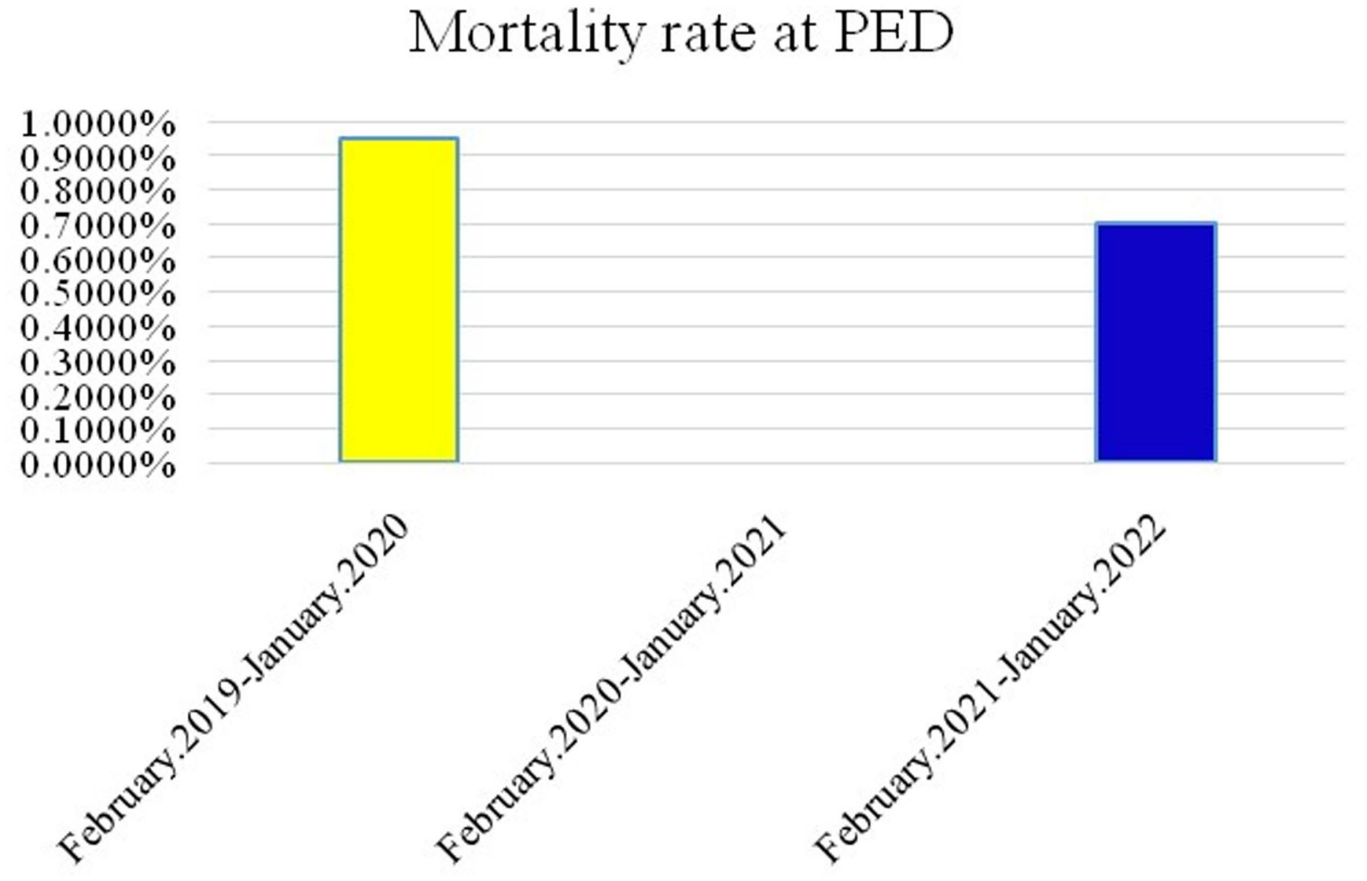
Mortality rate in children admitted to the ED in the 3 groups.

**Figure 3:**
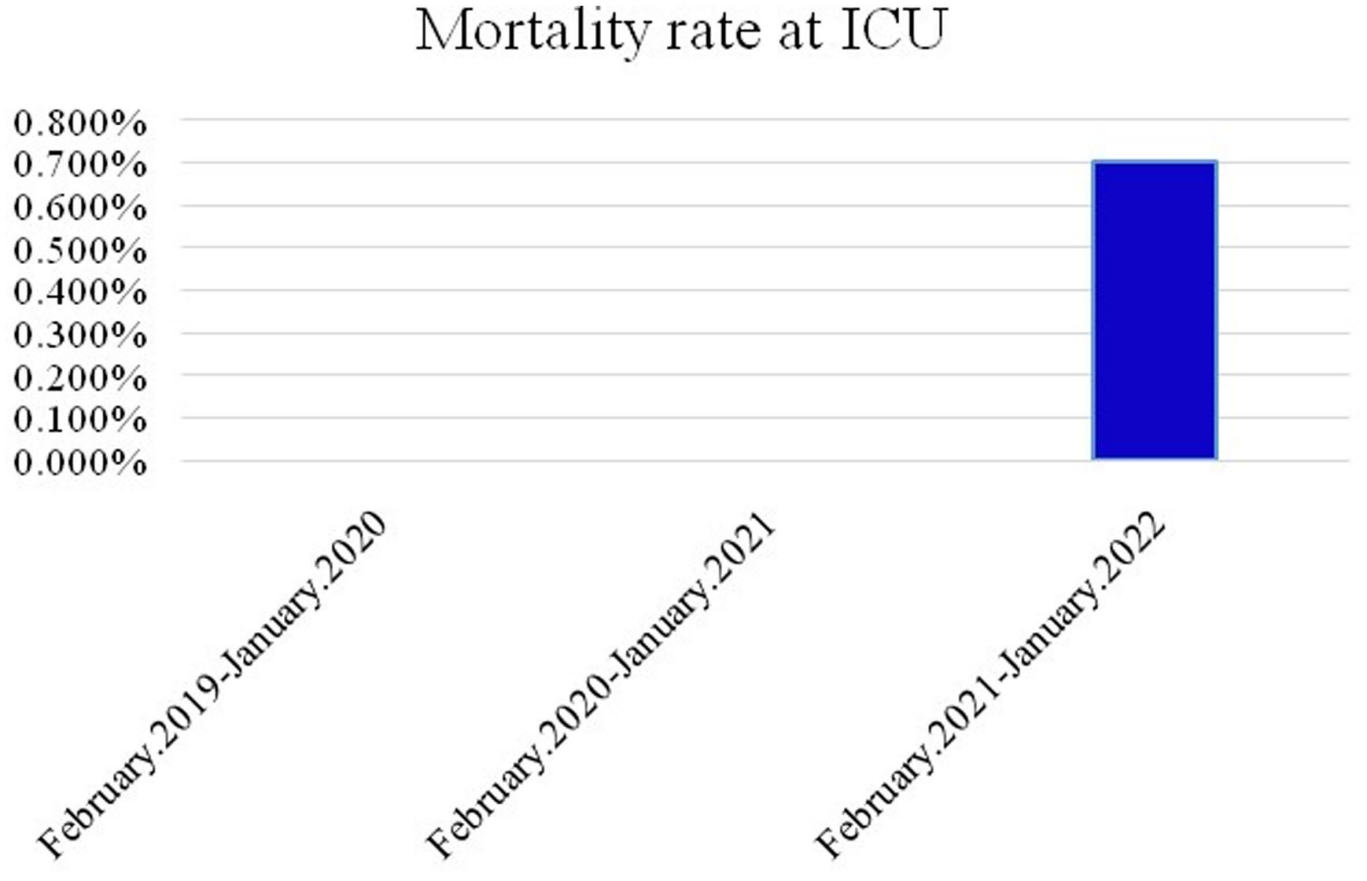
Mortality rate in children admitted to the ICUs in the 3 groups.

The ratio of sex, triage levels 1–3, and reasons for psychiatric consultation among the three groups were significantly different (*p* < 0.05). Elevated male female ratio is noted during and post pandemic period. Behavioral health problems were the main cause of psychiatric consultations in all three groups, with a larger number of females than males in each group. Age distribution revealed significant differences, with increasing numbers of middle childhood children (9–12 years) during the pandemic period. In addition, the number of behavioral health pediatric patients with suicidal ideation or suicide attempts sent to the ED increased about 5% during the pandemic period. The number of suicide attempts increased annually (Figure 1B), primarily due to drug overdose and self-inflicted injury, and showed significant differences among the three groups. Most common self-inflicted injury was cutting, whenever the COVID-19 outbreak or not. Notably, the number of jump-offs from buildings increased during the post pandemic period. In disposition, discharge was placement in patient safter visiting the ED in the three groups (*p* < 0.05), increasing annually. During the pandemic period, the rates of against-medical advice discharge (AAD) were at a three-year nadir, while the rate of hospitalization peaked. Most hospitalized patients were admitted to wards, and the mean length of general ward hospitalization in Group 2 was shorter than that in Groups 1 and 3 (*p* < 0.001). The length of hospitalization was significantly longer in Group 3 (IQR = 12.75) than in Group 2 (IQR = 5.5). Furthermore, ICU admission rates increased significantly after the COVID-19 outbreak. The mortality rates of patients admitted to the ED and ICU in the three groups are shown in Figures 2, 3, respectively. During the pandemic, no mortality occurred in either the ED or ICUs. However, in the post-pandemic period, the mortality rate increased in ICUs compared to that in the pre-pandemic and pandemic periods.

### Psychiatric diagnosis and substance use disorders in pediatric patients in the three groups

Major depressive disorder (MDD) accounted for most cases regardless of time period (Table 2). Furthermore, the incidence of MDD increased each year. Attention deficit hyperactivity disorder (ADHD) was the most common disorder in Group 2, which was four times higher than that in Group 1; adjustment disorder, schizophrenia, and psychosis had the lowest registrations in Group 2. Except for panic disorder, psychosis, anxiety, and obsessive-compulsive disorder (OCD), psychiatric disorders showed significant differences among the three groups (*p* < 0.05).

**Table 2:** Analysis of psychiatric diagnosis and drug abuses among the 3 groups.

Variables	Group 1	Group 2	Group 3	*p* for trend
	(*n* = 105)	(*n* = 93)	(*n* = 142)	
Psychiatric diagnosis				
MDD	23 (21.9)	30 (32.3)	48 (33.8)	<0.001***
Bipolar (Depression or mania)	15 (14.3)	6 (6.5)	23 (16.2)	0.0004***
Depression	13 (12.4)	16 (17.2)	21 (14.8)	0.0003***
Panic disorder	3 (2.9)	1 (1.1)	1 (0.7)	0.6701
ADHD	2 (1.9)	8 (8.6)	6 (4.2)	0.0132*
Adjustment disorder	5 (4.8)	4 (4.3)	9 (6.3)	0.013*
Schizophrenia	5 (4.8)	2 (2.2)	8 (5.6)	0.0308*
Psychosis	2 (1.9)	0 (0)	4 (2.8)	0.0763
Affective psychosis	0 (0)	0 (0)	3 (2.1)	0.0071**
Anxiety	8 (7.6)	7 (7.5)	7 (4.9)	0.2382
OCD	0 (0)	0 (0)	2 (1.4)	0.6739
Emotion problems	36 (34.3)	28 (30.1)	57 (40.1)	<0.001***
Types of drug				
BZD	18 (17.1)	31 (33.3)	28 (19.7)	<0.001***
TCA	0 (0)	1 (1.1)	3 (2.1)	0.0129*
SSRIs	12 (11.4)	18 (19.4)	12 (8.5)	0.0458*
SARI	1 (1.0)	1 (1.1)	1 (0.7)	0.5936
SNRIs	2 (1.9)	2 (2.2)	2 (1.4)	0.4504
Beta-adrenergic blocker	2 (1.9)	7 (7.5)	10 (7.0)	<0.001***
Buspirone	0 (0)	1 (1.1)	0 (0)	0.758
Acetaminophen	8 (7.6)	6 (6.5)	5 (3.5)	0.6276
NSAID	1 (1.0)	3 (3.2)	1 (0.7)	0.4909
Antihistamine	0 (0)	1 (1.1)	1 (0.7)	0.1878
Antipsychotics	11 (10.5)	17 (18.3)	17 (12.0)	0.0015**
Item of drugs				
1	13 (12.4)	15 (16.1)	13 (9.2)	0.0485*
2	11 (10.5)	12 (12.9)	13 (9.2)	0.0235*
3	8 (7.6)	14 (15.1)	4 (2.8)	0.553
4	3 (2.9)	5 (5.4)	2 (1.4)	0.5618
5	0 (0)	2 (2.2)	2 (1.4)	0.0625
6	0 (0)	0 (0)	5 (3.5)	0.0005***
10	0 (0)	0 (0)	1 (0.7)	–
11	0 (0)	1 (1.1)	1 (0.7)	–

The p-value is calculated by comparing the number of patients with psychological diseases to the total number of visits to the pediatric emergency department.

*p*-values, *p < 0.05; **p < 0.01; ***p < 0.001.

MDD, major depressive disorder; ADHD, attention deficit hyperactive disorder; OCD, obsessive-compulsive disorder; BZS, benzodiazepine; TCA, tricyclic antidepressant; SSRIs: selective serotonin reuptake inhibitors; SARI: serotonin antagonist and reuptake inhibitors; SNRIs: serotonin–norepinephrine reuptake inhibitors; NSAID, non- steroidal anti- inflammatory drugs.

**Figure 4:**
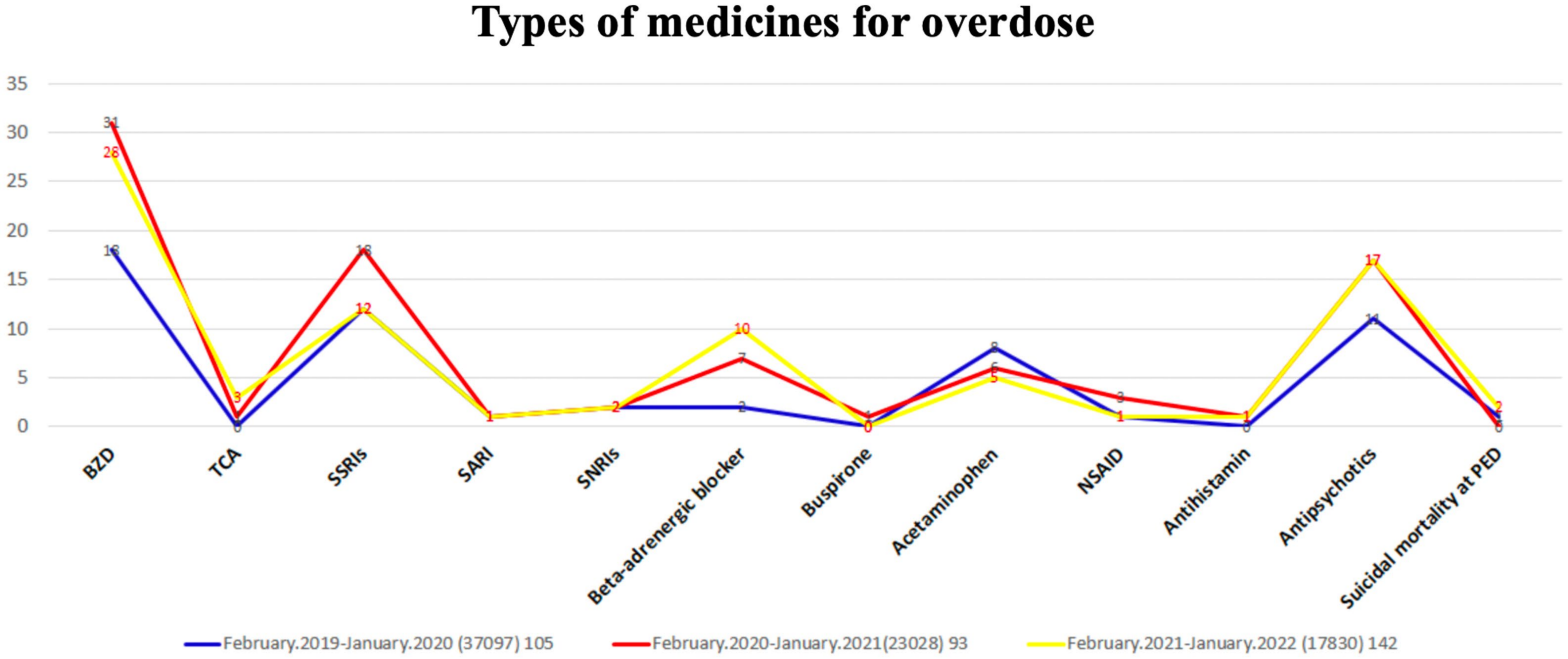
Types of medicine for overdose.

Benzodiazepine (BZD) was the most abused drug, followed by selective serotonin reuptake inhibitors (SSRIs), in children sent to the ED in the three groups (Figure 4). Overdoses of SSRIs peaked during the pandemic period. The rates of BZD, tricyclic antidepressant (TCA), beta-adrenergic blocker, and antipsychotic overdose showed a significant increase after the COVID-19 outbreak. Furthermore, most pediatric patients took one to three types of drugs, and the maximum drug use was 11 types of drugs.

## Discussion

Global public health strategies to limit the COVID-19 pandemic include enhanced hand hygiene, physical distancing, school and business closures, wearing of face masks, and restrictions on travel and social gatherings (Chiu et al., 2020). Epidemiological changes due to COVID-19 have led to a rapid decline in ED visits and hospitalizations (Ciofi degli Atti et al., 2020; Dopfer et al., 2020; Angoulvant et al., 2021; Chen et al., 2022). Although an epidemic prevention policy was developed to decrease the prevalence of infectious diseases after the pandemic (Chen et al., 2022), some psychiatric diseases have occurred frequently in children admitted to the ED in Taiwan. Children or adolescents in isolation, may also be at a risk of developing mental health problems (Singh et al., 2020). In China, prevalence of depression in children and adolescents increased by 9.1% during the pandemic period (Ma et al., 2021), while 16.3% of the respondents in the Philippines suffered from moderate or severe behavioral health impairment due to the pandemic (Tee et al., 2020). However, the impact of the COVID-19 on the epidemiological and clinical spectra of psychiatric problems in children admitted to the ED during the pandemic and post-pandemic periods has not been well surveyed. Our study revealed approximately 10% decline in pediatric ED visits due to psychiatric problems during the pandemic period, but a substantial increase in the post-pandemic period. This fluctuation is consistent with Centers for Disease Control and Prevention reports from the United States (Beaudry et al., 2022; Radhakrishnan, 2022). In our study, the proportion of ED visits caused by psychiatric problems increased during the pandemic period (0.4%) and the post-pandemic period (0.8%) compared to that in the pre-pandemic period (0.28%). This finding is consistent with an international study on pediatric ED visits (Krass et al., 2021).

Our study also revealed a higher rate of behavioral health problems due to the pandemic in female patients as compared to male patients visiting the ED. The rate of suicide in pediatric patients sent to the ED was slightly higher than that in the pre-pandemic period. This may be due to government prevention policy restrictions, disruptions in social relationships, and complicated mental health issues (Hill et al., 2021; Goto et al., 2022). Our results revealed an increase of approximately 10% in suicidal rate during the pandemic period, and further increase in the post pandemic period, from the pre pandemic period. The hospitalization rate due to psychiatric diseases increased during the pandemic period (33%) and the post-pandemic period (21.1%) compared to the in-pre-pandemic period. In addition, a higher ICU admission rate was noted during the pandemic period, indicating an increase in the severity of psychiatric diseases in patients admitted to the ED. Moreover, the proportion of psychosomatic pediatric patients in the ED triaged as levels 1 and 2 showed a significant increase; however, the proportion of levels 3 and 4 patients decreased after the COVID-19 outbreak. A higher proportion of triage levels 1 and 2 may indicate the severity of pediatric patients requiring further ICU critical care. Some studies have also shown that ICU admission rate is strongly associated with ED triage levels (Gravel et al., 2009). This may explain the higher rate of ICU admissions in our study. The mean length of ward hospitalization and observational time in the ED decreased for children with psychiatric disorders during the pandemic period compared to that in the pre-pandemic and post-pandemic periods. The length of hospital stay in children with psychiatric diseases in both the pediatric ED and wards may have decreased owing to the potential fear of COVID-19 infection in the hospitals (Fasshauer et al., 2021).

Since the present study showed that suicide attempts in overall pediatric ED visits increased during the COVID-19 period and further increased in the post-pandemic period, while suicidal ideation was more common in the post-pandemic period than before and during the pandemic, it is important to prevent suicide attempts and suicidal ideation in the post-pandemic period. Therefore, governments must establish policies to address this critical issue. Moreover, considering that cutting and jumping were identified as the primary methods of suicide injury, and that BZDs, SSRIs, and antipsychotics were the most commonly overdosed prescription drugs, either in overdose attempts or obtained from others, we believe that these findings have the potential to contribute to the prevention and mitigation of suicide attempts among children and adolescents. Before the pandemic, anxiety, behavior, and mood disorders were the top three mental disorders among children (Merikangas et al., 2009). After the COVID-19 outbreak, an increase in anxiety and depression symptoms and a decrease in life satisfaction among adolescents have been noted (Magson et al., 2021). Another study had unveiled the prevalence rates of clinically elevated depression and anxiety symptoms in children and adolescents on a global scale (Racine et al., 2021). Additionally, it found that children and adolescents experienced worsened anxiety and depression levels following the COVID-19 pandemic (Wang et al., 2022). In the present study, an increase in the proportion of patients with MDD and depression was observed during the pandemic. Moreover, children or adolescents with neurodevelopmental disorders may be at a higher risk of experiencing psychological fragility. Those with ADHD exhibited an increased prevalence of exaggerated startle responses, difficulties in waking up, angry moods, and COVID-19-related fears compared to typically developing children and adolescents (Giallonardo et al., 2021). Another study also revealed a higher likelihood of negative emotional and behavioral changes in children aged 1–6 years with autism spectrum disorder (ASD) (Zhao et al., 2023). Mental problems caused by the COVID-19 pandemic in adolescents may have serious and lasting effects, leading to poor mental and physical health conditions (Jones et al., 2021). Therefore, it is important to support and help children and adolescents, whether they have underlying neurodevelopmental disorders or not, by taking out time to connect them with friends and family.

This study had certain limitations that should be acknowledged. First, due to its retrospective design, it was challenging to identify pediatric patients with underlying psychological or neurodevelopmental disorders. Secondly, the study did not evaluate the potential protective factor of psychotherapy and counseling interventions, which individuals often utilize without a prescription to prevent or alleviate symptoms at the onset. Thirdly, previous psychiatric disorders, especially neurodevelopmental disorders, are a frequent comorbidity in subjects who do an ED admission for psychological fragility. Moreover, subjects with ASD and ADHD showed worrying symptomatology during the first phases of COVID-19 pandemic (Giallonardo et al., 2021; Zhao et al., 2023), thus suggesting that a cluster of subjects in our sample could have a neurodevelopmental disorder and that this could be an important variable that should have been considered in our analyses. By the way, due to the retrospective nature of this study, it was impossible including a cluster analysis on these subjects. Finally, the study was conducted in a single medical center in Taiwan, which may limit the generalizability of the findings.

## Conclusion

Our study revealed an increase in the proportion of pediatric psychiatric patients during the COVID-19 pandemic and post-pandemic period in the ED. Higher ICU admission rates and increased types of substance use disorders were noted during the two-year pandemic period. Most importantly, suicide attempts in children and adolescents were more common in the pandemic period than before and post the pandemic. It is crucial to provide mental health support to children and adolescents to prevent suicide attempts and drug overdose in the event of a new global outbreak of infectious diseases.

## Data availability statement

The original contributions presented in the study are included in the article/supplementary material, further inquiries can be directed to the corresponding authors.

## Ethics statement

The studies involving humans were approved by the Institutional Review Board of China Medical University Hospital (CMUH110-REC3-083). The studies were conducted in accordance with the local legislation and institutional requirements. Written informed consent for participation was not required from the participants or the participants’ legal guardians/next of kin because this is retrospective study. All methods were performed in accordance with the relevant guidelines and regulations. Data were collected, reviewed, de-identified, and anonymized before analysis. The ethics committee waived the requirement for informed consent because of the anonymized nature of the data and the scientific purpose of the study.

## Author contributions

B-CG: Data curation, Formal analysis, Supervision, Writing – original draft. Y-JC: Data curation, Formal analysis, Investigation, Methodology, Writing – original draft. W-YH: Formal analysis, Investigation, Writing – original draft. M-JL: Methodology, Supervision, Writing – original draft. H-PW: Conceptualization, Investigation, Supervision, Writing – review & editing.

## Abbreviations

COVID-19: Coronavirus disease 2019
MDD: Major depressive disorder
ADHD: Attention deficit hyperactive disorder
OCD: Obsessive-compulsive disorder
OHCA: Out-of-hospital cardiac arrest
PED: Pediatric emergency department
ICU: Intensive care unit
ROSC: Return of spontaneous circulation
IQR: Interquartile range
MBD: May be discharge
AAD: Against advice discharge
